# Synergistic Antitumor Effects on Drug-Resistant Breast Cancer of Paclitaxel/Lapatinib Composite Nanocrystals

**DOI:** 10.3390/molecules25030604

**Published:** 2020-01-30

**Authors:** Jun Wang, Feng-Mei Lv, Dong-Li Wang, Jian-Liang Du, Hai-Yan Guo, Hai-Ni Chen, Shou-Jin Zhao, Zhe-Peng Liu, Yu Liu

**Affiliations:** 1 School of Medical Instrument and Food Engineering, University of Shanghai for Science and Technology, Shanghai 200093, China; wangjun245186@126.com (J.W.); LFMusst@126.com (F.-M.L.); HN_C383@163.com (H.-N.C.); shoujinzhao@163.com (S.-J.Z.); 2 Department of Pharmaceutics, School of Pharmacy, Fudan University & Key Laboratory of Smart Drug Delivery (Fudan University), Ministry of Education, Shanghai 201203, China; dlwang13@fudan.edu.cn (D.-L.W.); hyguo829@126.com (H.-Y.G.); liuyu@fudan.edu.cn (Y.L.); 3 Department of Chinese Medicine, Shanghai University of Traditional Chinese Medicine, Shanghai 201203, China; dujianliang01@126.com

**Keywords:** paclitaxel, lapatinib, composite nanocrystal, synergy, drug-resistant

## Abstract

Drug resistance presents serious difficulties for cancer treatment. A combination of paclitaxel (PTX) and lapatinib (LAPA) shows potentials in multiple drug resistant cancers in the clinic, but it is almost impossible to deliver these two drugs to the tumor at the same time with the best proportion by simple co-administration of the respective current formualtions for their different pharmacokinetic profiles. Here composite nanocrystals of PTX and LAPA (cNC) were designed with a ratio of 2:1 (*w*/*w*), which was their intracellular ratio at the best synergistic efficacy on a drug-resistant cancer cell line (MCF-7/ADR). Such cNC were prepared using a bottom-up method to achieve a nearly spherical appearance and a narrow size distribution of 95.1 ± 2.1 nm. For nanocrystal stabilization, Polyethylene glycol (PEG) coating was introduced into the cNC via polydopamine (PDA) coating in order to get a PEGylated composite nanocrystal (cNC@PDA-PEG) with nanoscale size (170.5 ± 1.4 nm), considerable drug loading (PTX: 21.33 ± 1.48%, LAPA: 10.95 ± 1.24%) and good stability for at least 4 days in plasma-containing buffers. Differential scanning calorimeter (DSC) and XRD data both indicated the different crystalline states of the cNC as well as the cNC@PDA-PEG in comparison with bulk drugs. In vitro release data showed that PTX and LAPA were gradually and completely released from cNC@PDA-PEG in 3 days, while drug release from bulk drugs or cNC was only 30%. cNC@PDA-PEG also showed negligible hemolysis in vitro. Cellular uptake experiments in the MCF-7/ADR cell line showed that the nanocrystals entered the cells in a complete form through endocytosis and then released the drug in the cell. cNC@PDA-PEG inhibits the growth of this drug-resistant cell more effectively than the unmodified version (cNC). In summary, PEGylated PTX and LAPA composite nanocrystals showed the potential for treament of drug-resistant tumors by simultaneously delivering two drugs to tumor cells with the best proportion.

## 1. Introduction

Drug resistance of cancer cells is one of the main reasons for the failure of cancer treatment, which may lead to rapid recurrence or disease progression of cancer and result in death [1,2,3]. For example, breast cancer is the first cause of death in women. Most patients with breast cancer respond well to chemotherapy at first, but gradually develop resistance during chemotherapy. Such resistance to chemotherapeutic drugs is believed to be related with the overexpression of ABC (ATP binding cassette) transporters [4,5].

Paclitaxel (PTX) leads to mitotic arrest and apoptosis by stabilizing the microtubule structure in cells [6]. It has been used in the treatment of various types of solid tumors, but the response rate of PTX alone is not satisfactory [7,8,9,10]. Lapatinib (LAPA) is a small casein kinase inhibitor of EGFR (epidermal growth factor receptor) and HER2 (human epidermal growth factor receptor-2) and a p-glycoprotein inhibitor. However, LAPA alone showed limited efficacy [11,12,13,14]. In clinical trials, PTX and LAPA are often used in combination [15]. In 2008 and 2013, two phase III studies in HER2-positive metastatic breast cancer showed that the combination of PTX and LAPA leads to better survival than PTX alone [16,17]. Another phase III study in 2014 also showed that PTX plus LAPA had a better anti-tumor effect [18]. Inspired by these clinical studies, PTX and LAPA were given in combination. But it is almost impossible to deliver these two drugs to the tumor at the same time with the best proportion by a simple combination of the respective current formualtions because each drug has its own unique pharmacokinetic profiles [19,20]. But when drugs are coencapsulated in the same nano-carrier, the exposure of both drugs will be more controlled, which is beneficial for their synergistic effect [21]. In 2016, Goldman designed nanoparticles with “2-in-1” drugs which showed better anti-tumor effect than single drug-loaded nanoparticles and could be expected to assist synergistic actions between drugs to overcome drug resistance [22].

Several teams have reported new codelivery systems for LAPA and PTX [23,24,25,26]. However, these drug delivery systems used a large proportion of polymer materials to constitute the carrier, limiting drug loading. Nanocrystals may provide an alternative form to achieve good aqueous dispersity and high drug content for poor water-soluble drugs [27]. As “pure drug particles”, in theory, they require only a small amount of surfactants as stabilizers. In addition, their preparation is simple and easy to scale up [28]. However, there are few reports about composite nanocrystals composed of two chemotherapeutic drugs.

As each coin has two sides, nanocrystals also have disadvantages [29]. Stability is the key factor to ensure the safety and effectiveness of nanocrystals, which need close monitoring in production and storage. For stabilization, surface modification of nanocrystals is indispensible. Most previous modifications were based on physical adsorption [30,31], which was relatively unstable and cannot withstand long-term storage and complex in vivo environments.

Recently, we got some inspiration from mussels in the ocean for surface modification of nanocrystals [32]. Mussels rely on the repetitive structure of levodopa in their foot filaments to stick firmly to the bottom of the ship [33,34,35,36]. Further studies found that dopamine can undergo self-polymerization under alkaline conditions and form polydopamine (PDA) similar to the components in mussel foot filaments. When solid materials are immersed in the aqueous solution of dopamine, dopamine polymerizes firmly adsorb on the surface of the solid material [37]. We found that nanocrystals can also act as the solid material on which dopamine polymerized to form PDA coating [32]. Moreover, such a polydopamine film contains active catechol hydroxyl groups, which can further react with amino groups under mild conditions [38], which provided a good active reaction platform for functional modification of nanocrystals. Here we used this activity of PDA coating for PEGylation of nanocrystals for stabilization and long circulation functionalization of nanocrystals.

**Scheme 1:**
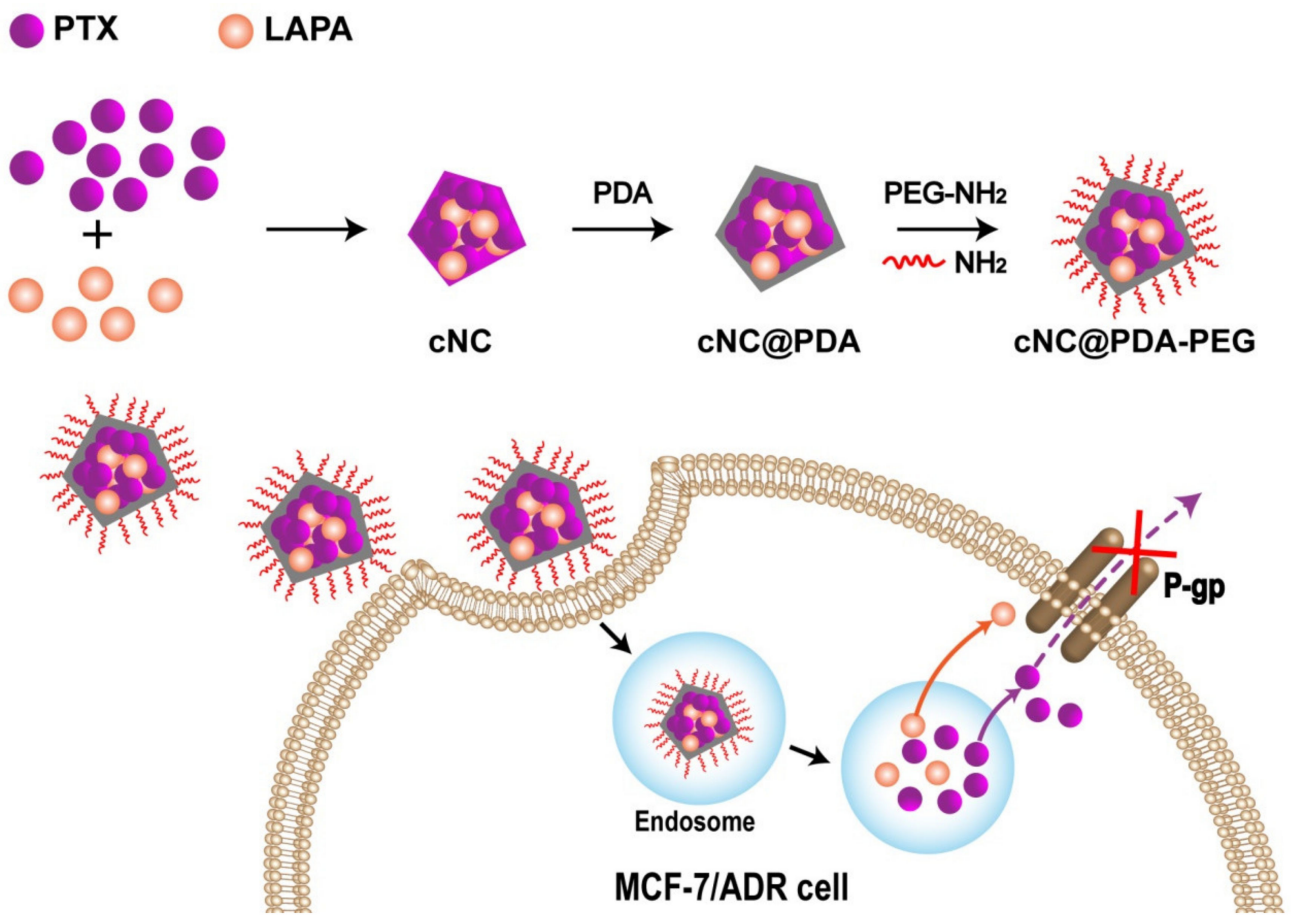
Illustration of the treatment of multidrug-resistant cancer with the cNC@PDA-PEG. PTX: paclitaxel; LAPA: lapatinib; cNC: composite nanocrystals; PDA: polydopamine; PEG: Polyethylene glycol.

In this study, PTX and LAPA composite nanocrystals with PDA and PEG modification (cNC@PDA-PEG) were designed and prepared successfully (Scheme 1). The appropriate ratio of PTX and LAPA was optimized. cNC@PDA-PEG had a better therapeutic effect on a drug-resistant cancer cell line (MCF-7/ADR) compared to a simple mixture of PTX and LAPA. This delivery system provides a platform for synergic delivery of PTX and LAPA for the chemo-resistant breast tumor.

## 2. Results and Discussion

### 2.1. Optimization

**Figure 1:**
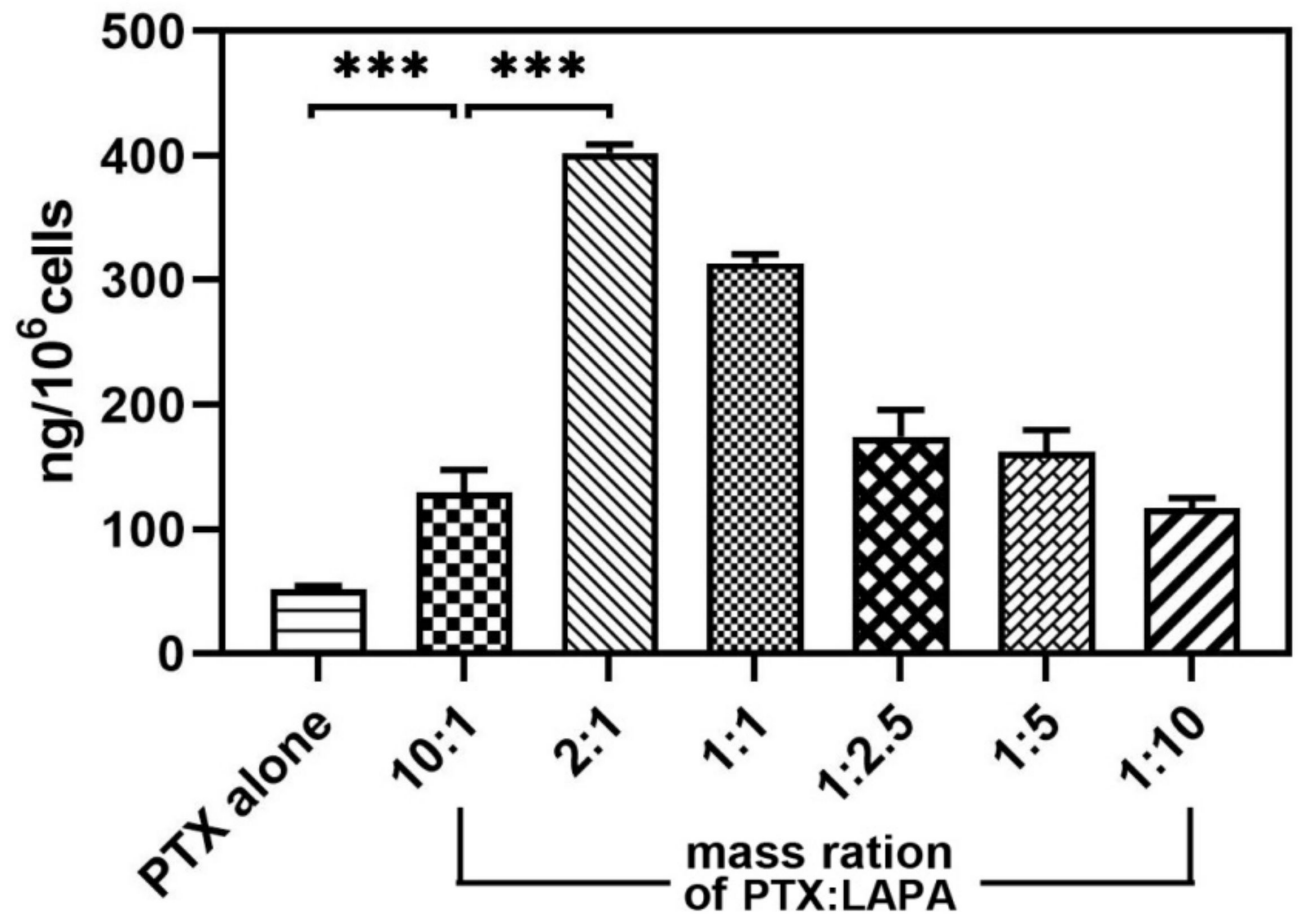
Intracellular paclitaxel content after incubation of the free drug (paclitaxel vs. paclitaxel plus lapatinib at different ratios) for 4 h in MCF-7/ADR cells (n = 3, mean ± SD, *** *p* < 0.001).

The drug resistance of the MCF-7/ADR cell line was verified by a resistance index (RI) value before the cytotoxicity experiment. The value of RI was 24.58, which indicated that MCF-7/ADR was resistant to PTX (Figure S1). The results of intracellular concentration (Figure 1) showed that when only PTX was given at 0.075 mg/mL, the amount of intracellular PTX was 52.27 ng per 10^6^ cells, and after adding LAPA with 0.0075 mg/mL, the amount of intracellular PTX was 129.56 ng per 10^6^ cells, which was 2.48 times higher than that of the PTX group alone. With the increase of the concentration of LAPA, the amount of intracellular PTX increased. When the concentration of LAPA was 0.0375 mg/L, the intracellular amount of PTX was 402.30 ng per 10^6^ cells. However, when the amount of LAPA was equal to or more than that of PTX, the amount of intracellular PTX decreased. Results of MTT (Table 1) showed that when the ratio of LAPA was increased, CI_50_ decreased from 0.23 to 0.14, indicating that without the assistance of LAPA, PTX alone could not be effective for MCF-7/ADR cells to overcome their resistance to PTX. However, with a further increase of LAPA, the CI_50_ increased from 0.22 to 2.07, which was consistent with the intracellular concentration results that the amount of intracellular PTX decreased when the ratio of LAPA exceeded PTX. It was clear that an appropriate ratio of PTX and LAPA was needed to overcome the drug resistance of cells, and excessive LAPA would not benefit the cytotoxicity of PTX. It was of ultimate importance to control the mass ratio of PTX and LAPA that entered cells at 2:1.

### 2.2. Successful Preparation of cNC@PDA-PEG

**Figure 2:**
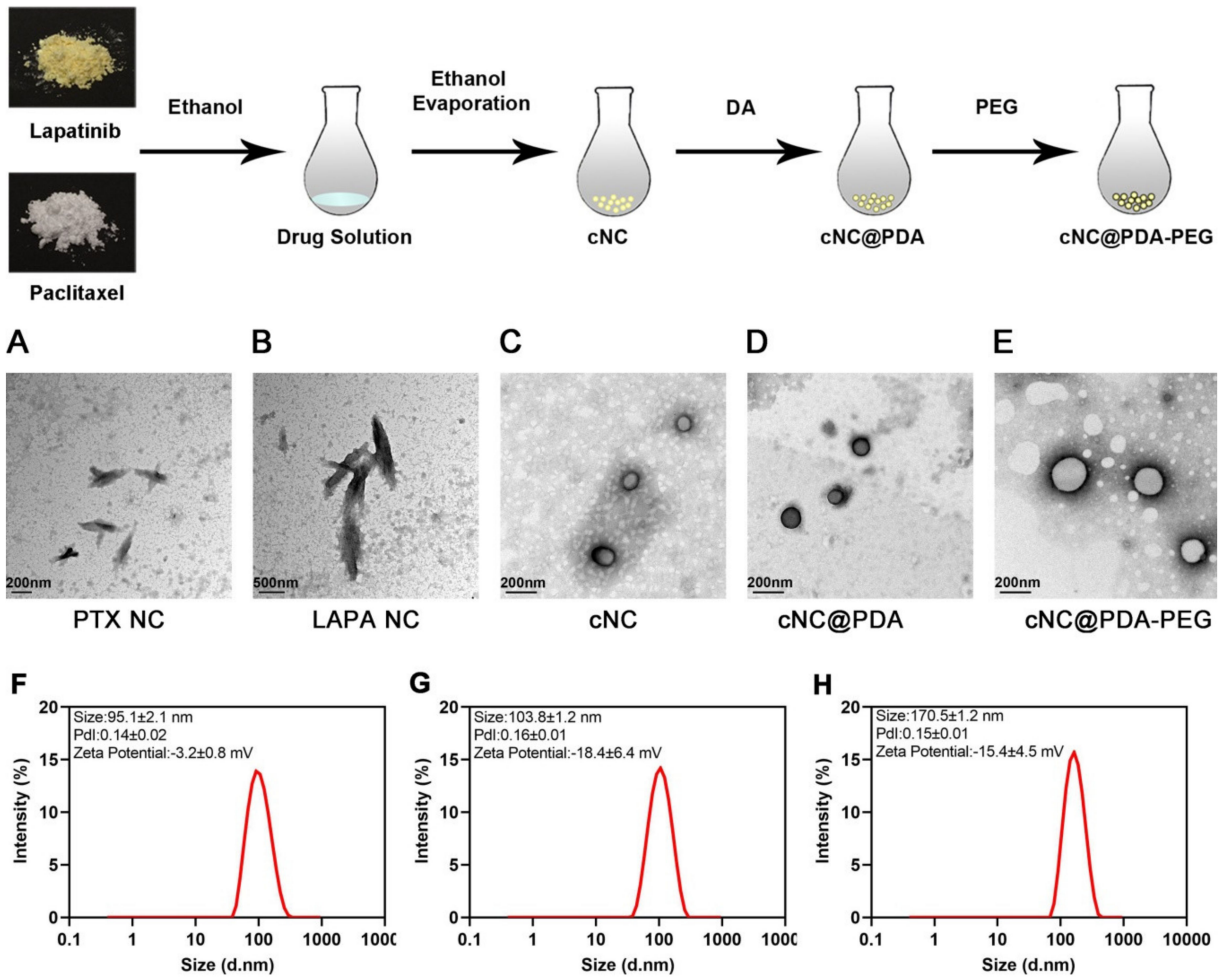
Characterization of cNC, cNC@PDA, and cNC@PDA-PEG: (**A**–**E**), TEM images; (**F**–**H**), size distribution and Zeta-potential measured by dynamic light scattering (DLS).

cNC of PTX and LAPA with the most effective mass ratio 2:1 were successfully prepared by a bottom-up thin film hydration method. More formulation optimization is described in the Supplementary Materials (Table S1, Figure S2). cNC was formed by hydration of thin films. Subsequently, dopamine was added to self-polymerize on the surface of the cNC in order to obtain cNC@PDA. As the PDA layer might continue to self-polymerize, resulting in further polymerization between nanocrystals, the surface of the PDA was modified by PEG. MeO-PEG3000-NH_2_ was added to react with the PDA coating. Excess MeO-PEG3000-NH_2_ was removed by centrifugation to obtain cNC@PDA-PEG (Figure 2). As shown in Table 2, the DL of PTX and LAPA in the cNC were 32.35 ± 1.48% and 15.31 ± 1.57%, respectively; after PDA and PEG treatment, the DL of PTX and LAPA in the cNC@PDA were 25.38 ± 1.56% and 13.33 ± 1.43%, respectively, and the DL of PTX and LAPA in cNC@PDA-PEG were 21.33 ± 1.43% and 10.95 ± 1.24%, respectively. The DL ratio of PTX to LAPA measured in cNC, cNC@PDA and cNC@PDA-PEG was close to 2:1. The total DL of PTX and LAPA after PEGylated was more than 30%, which has the advantage of high drug loading compared with previous studies [23,24,25,26].

As shown in Figure 2, cNC, cNC@PDA and cNC@PDA-PEG all exhibited a near-spheroid shape, different from PTX NC and LAPA NC, which tend to form needle-like crystals. Needle-like nanocrystals have poor stability and difficulty in functionalization, so it is necessary to improve their morphology. The size of cNC, cNC@PDA and cNC@PDA-PEG were 95.1 ± 2.1 nm, 103.8 ± 1.2 nm and 170.5 ± 1.4 nm, respectively. The zeta potential of cNC, cNC@PDA and cNC@PDA-PEG were −3.2 ± 0.8 mV, −18.4 ± 6.4 mV and −15.4 ± 4.5 mV, respectively.

**Figure 3:**
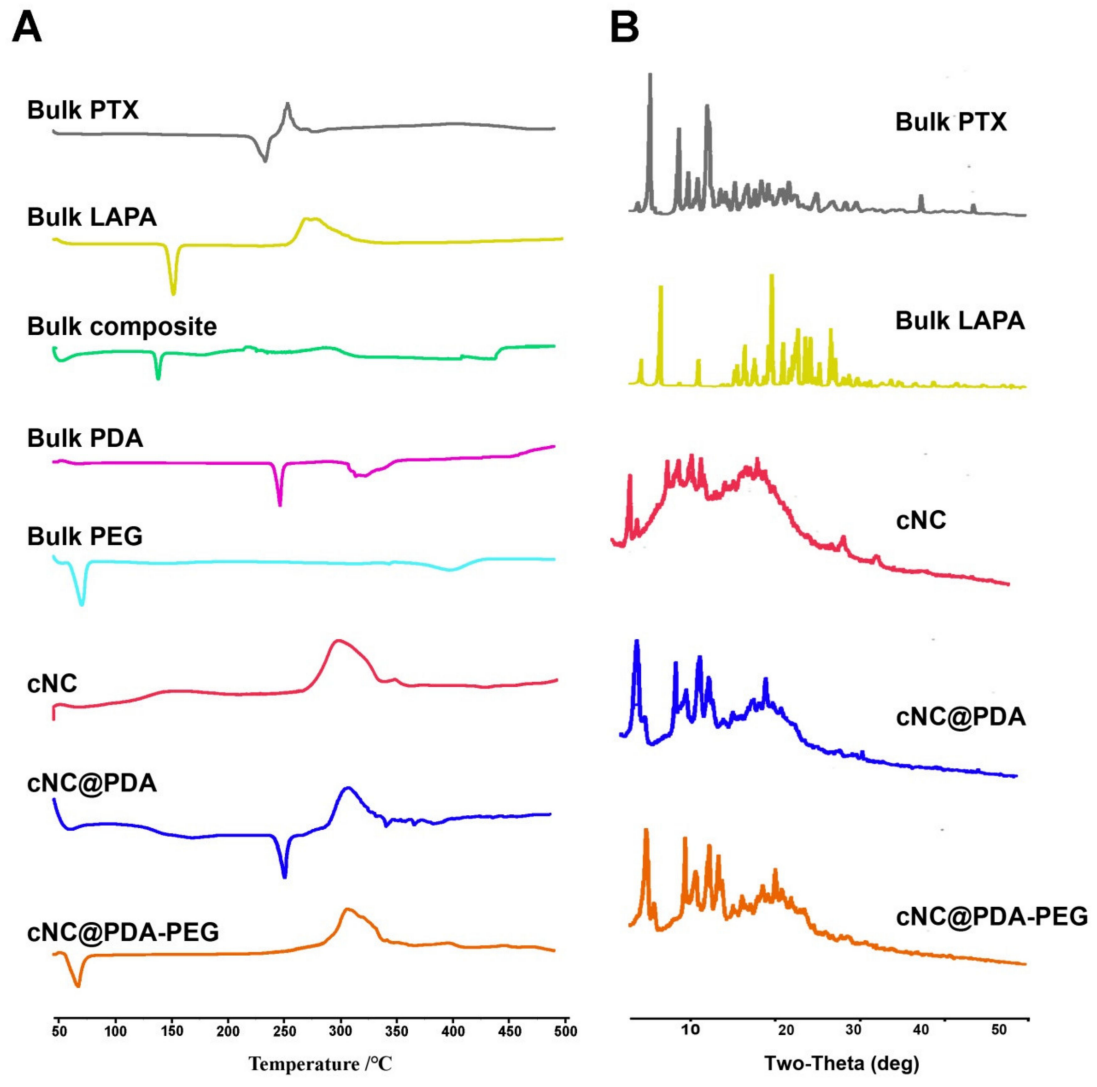
(**A**) Differential scanning calorimeter (DSC) and(**B**) X-ray Powder Diffraction (XRPD) analysis of cNC, cNC@PDA and cNC@PDA-PEG.

DSC curves of various bulk drug and nanocrystals are shown in Figure 3A. Bulk paclitaxel showed an exothermic peak at 232 ℃ and an endothermic peak at 253 ℃. Bulk lapatinib showed an exothermic peak at 152 ℃ and an endothermic peak at 275 ℃. However, the spectra of cNC, cNC@PDA and cNC@PDA-PEG did not show the above two characteristic peaks. They all showed another exothermic peak at 298 ℃, indicating a different crystalline state. cNC@PDA-PEG showed no peak at 250 ℃ compared with cNC@PDA but an exothermic peak at 67 °C, which is similar to bulk PEG, indicating that cNC was coated on PEG. The XRPD patterns of bulk PTX displayed obvious peaks at 5.6°, 9.0° and 12.0°, the patterns of bulk LAPA displayed obvious peaks at 6.8°, 20.0°, 23.0° and 27.0°, and the patterns of cNC, NC@PDA and NC@PDA-PEG showed distinct peaks at 5.2°, 9.7° and 12.5°, respectively, also indicating a different crystalline from bulk drug (Figure 3B).

**Figure 4:**
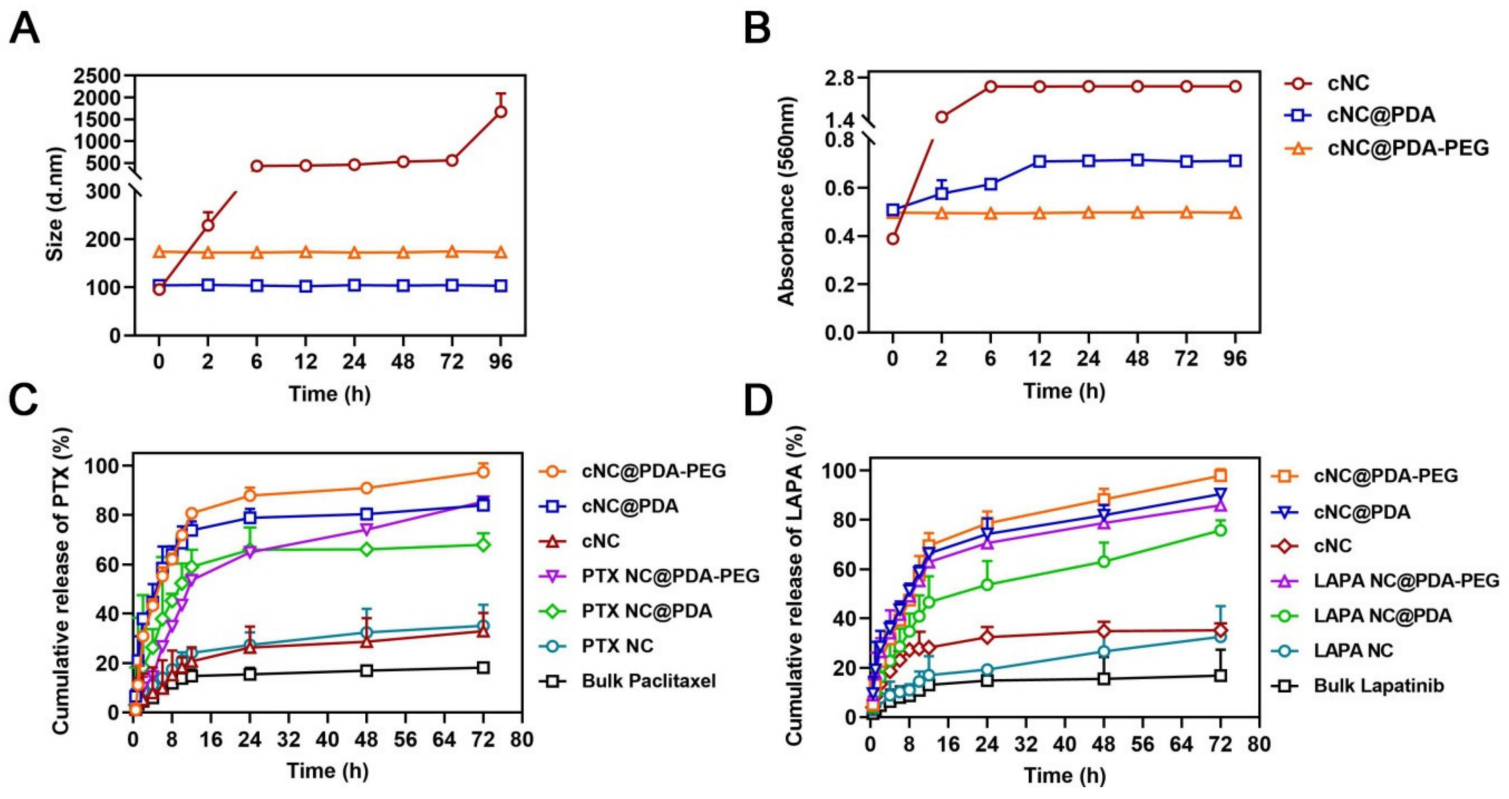
Stability and release profiles of different composite nanocrystals. (**A**) Size change in PBS measured by DLS. (**B**) Size aggregation in 10% fetal bovine serum (FBS) measured by absorbance at 560 nm. In vitro release profiles of PTX (**C**) and LAPA (**D**) from different formulations in PBS by the dialysis method (n = 3, mean ± SD).

The particle size change of different composite nanocrystals in PBS during storage (Figure 4A) was measured by DLS, and the particle aggregation of different composite nanocrystals in PBS and 10% FBS was evaluated by absorbance at 560 nm (Figure S3 and Figure 4B). The results showed that cNC aggregated quickly in PBS and 10% FBS, while cNC@PDA and cNC@PDA-PEG were stable in both media for a long time. The release of PTX from cNC@PDA-PEG was the fastest and that from bulk paclitaxel was the slowest. The cumulative release of PTX and LAPA from cNC@PDA-PEG reached 97.41% and 97.84% at 72 h, respectively, while both were less than 40% in cNC and the bulk drug. The release from cNC and the bulk drug was the slowest because they both precipitated and clumped in the dialysis bag. Moreover, the release performance of PTX from cNC and PTX NC was similar, indicating that the additional LAPA had little effect on the release performance of PTX in cNC.

### 2.3. Hemolysis Assay

**Figure 5:**
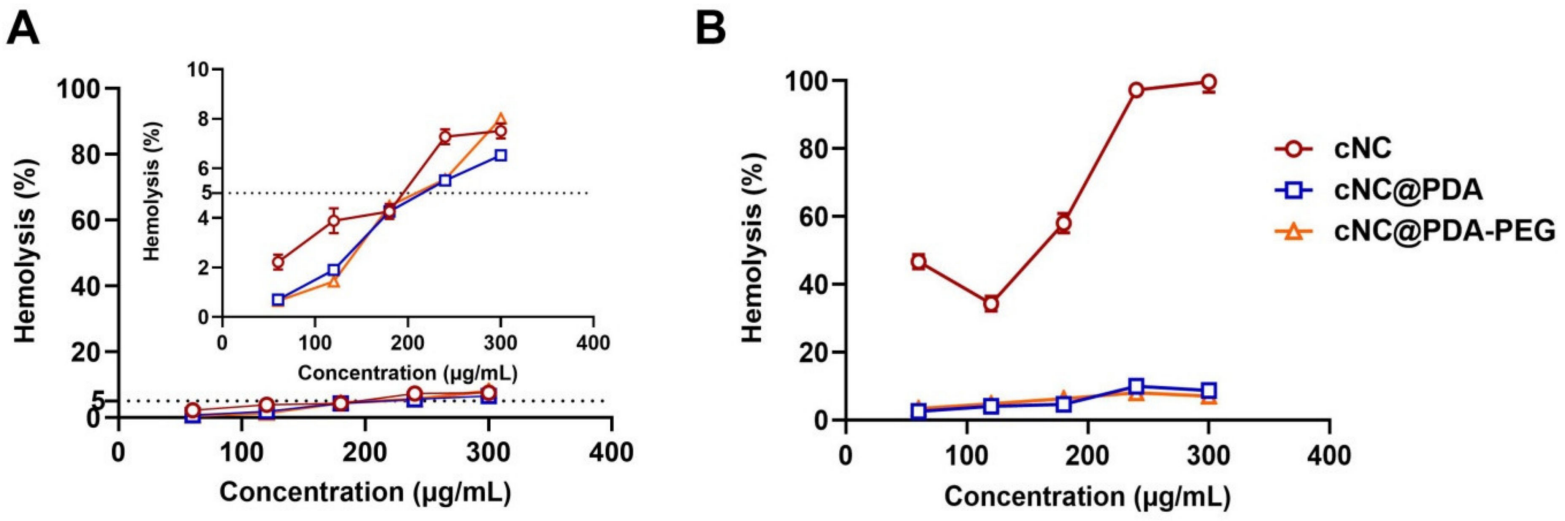
Hemolysis induced by cNC, cNC@PDA and cNC@PDA-PEG: (**A**) 2 h; (**B**) 6 h (n = 3, mean ± SD).

Blood compatibility is one of the important indexes of safety evaluation. Therefore, the effects of different nanocrystals on the red blood cell (RBC) membrane were investigated. The hemolysis rates of different nanocrystals were less than 8.5% at 2 h, with no obvious hemolysis observed (Figure 5A). At 6 h, the hemolysis rate of cNC was as high as 99.67% with a concentration of 300 μg/mL, indicating that the structure of the RBC membrane was greatly damaged, resulting in severe hemolysis. However, the percentage of hemolysis in the cNC@PDA and cNC@PDA-PEG groups at different concentrations was less than 9%, which was negligible compared with the positive control group (Figure 5B). The results showed that PEG had good blood compatibility and was safe.

### 2.4. Enhanced Cellular Uptake and Cytotoxicity of Nanocrystals by PEG Modification

**Figure 6:**
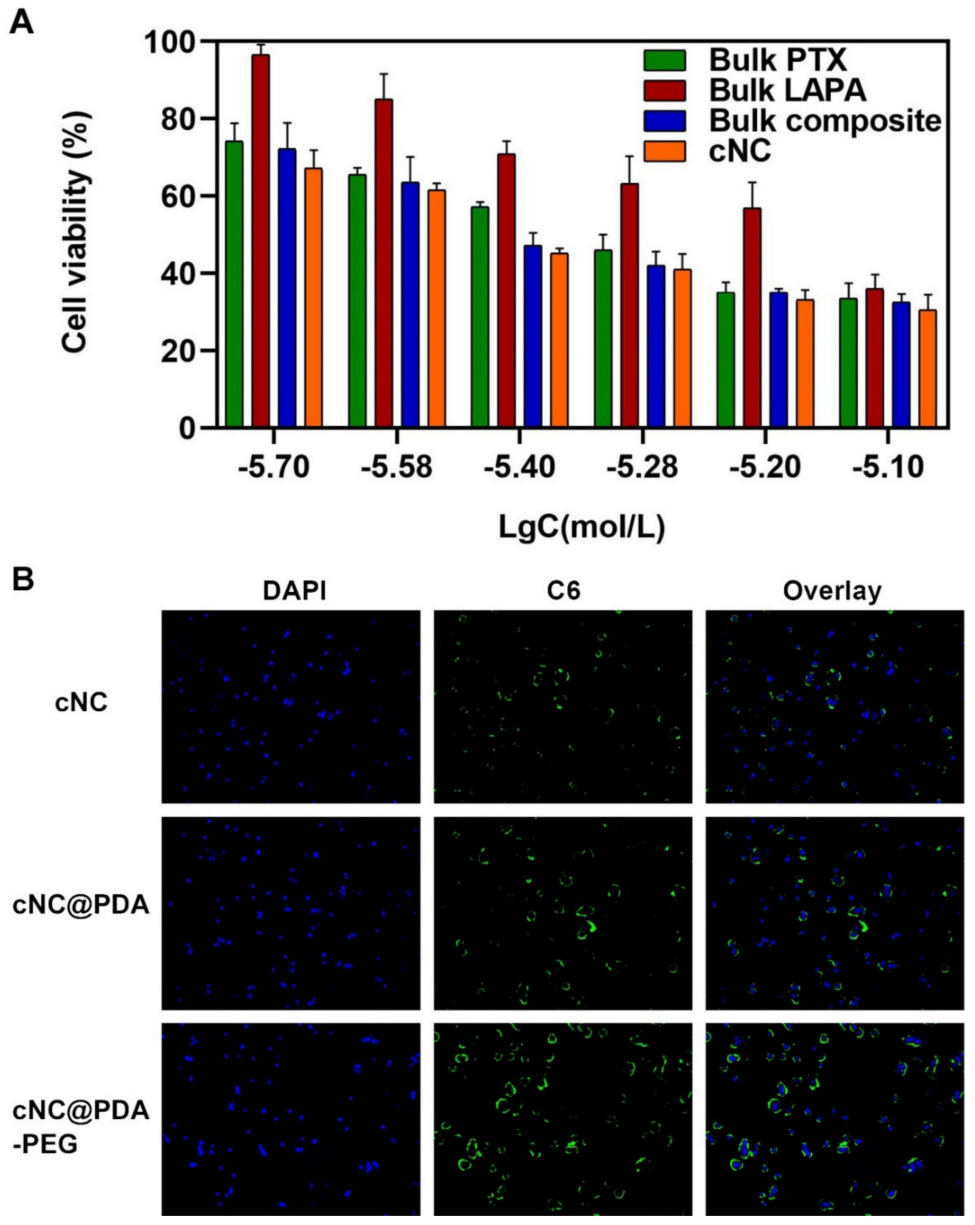
(**A**) In vitro cytotoxicity of bulk drug and composite nanocrystals after 24 h co-incubation (n = 3, mean ± SD). Tested cell line: Drug-resistant human breast cancer cells MCF-7/ADR. (**B**) Fluorescent microscopic photographs of MCF-7/ADR cells after incubation for 4 h with C6-labeled cNC, cNC@PDA and cNC@PDA-PEG.

Cytotoxicity was evaluated at different concentrations of bulk PTX, bulk LAPA, bulk composite and cNC. The results showed that the cNC showed stronger anti-proliferative activity (Figure 6A), which might benefit from the fact that the cNC contributes to the co-delivery of drugs and was more conducive to the destruction of drug resistance. Cellular uptake of different composite nanocrystals was tested with MCF-7/ADR cells. More cNC@PDA-PEG was taken up than cNC and cNC@PDA (Figure 6B), perhaps benefiting from its better stability.

**Figure 7:**
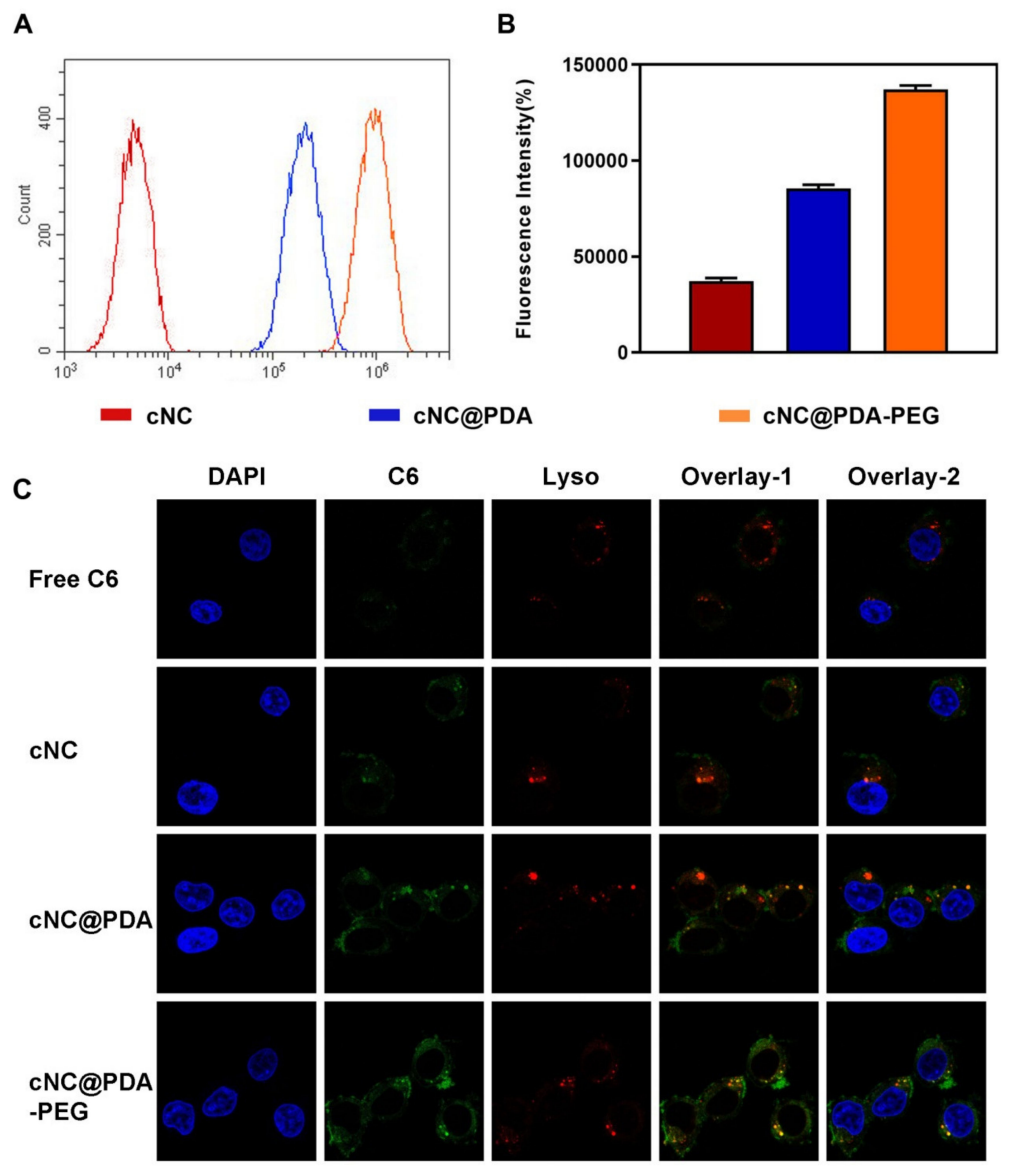
(**A**) Fluorescent intensity of cells after co-incubation for 4 h with C6-labeled composite nanocrystals measured using flow cytometry. (**B**) Average fluorescence intensity of figure (A) (n = 3, mean ± SD). (**C**) Confocal images for intracellular localization of different composite nanocrystals in MCF-7/ADR cells after 4 h incubation. The nucleus was stained with DAPI (blue) and the lysosomes were stained with LysoTracker Red.

After MCF-7/ADR cells were incubated with different composite nanocrystals for 4 h, the uptake of cNC@PDA-PEG was higher than that of other groups, which was shown in flow cytometry (Figure 7A) and average fluorescence intensity (Figure 7B). In order to investigate the uptake process of different composite nanocrystals, MCF-7/ADR cells were incubated with cNC, cNC@PDA and cNC@PDA-PEG for confocal observation after lysosome labeling (Figure 7C). It was obvious that more cNC@PDA and NC@PDA-PEG were taken up by MCF-7/ADR. The fluorescence of cNC@PDA-PEG was close to the nucleus and overlapped with the fluorescence of lysosome, indicating possible involvement of an endocytosis pathway.

**Figure 8:**
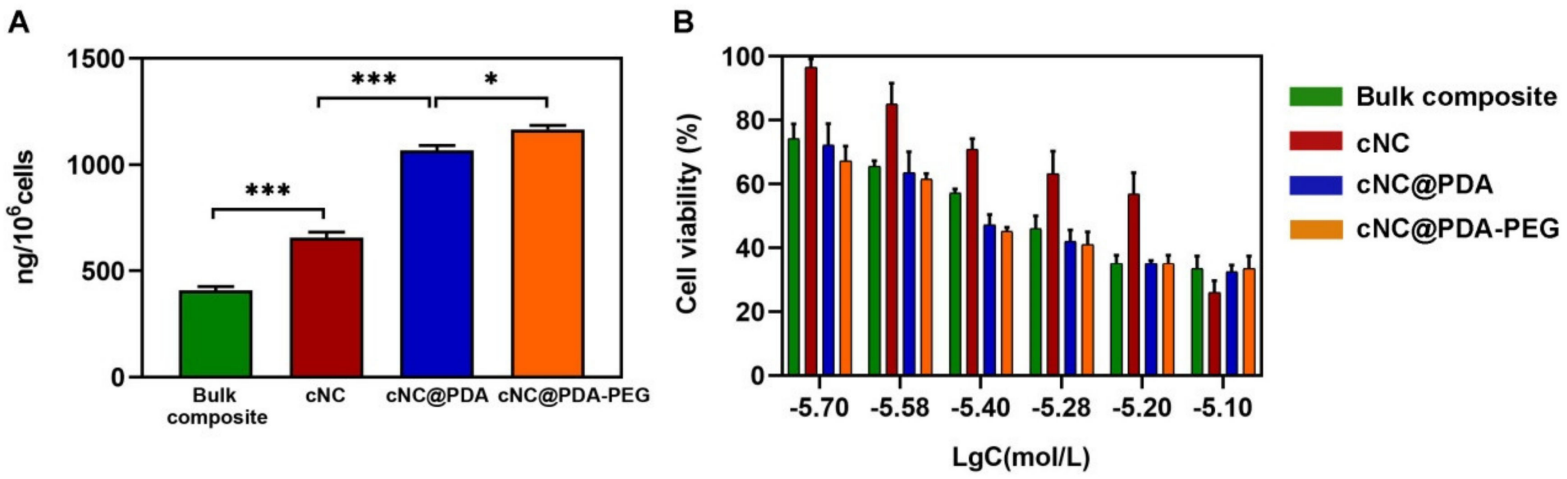
(**A**) Intracellular paclitaxel content after co-incubation for 4 h with bulk composite or different composite nanocrystals (n = 3, mean ± SD, * *p* < 0.05, *** *p* < 0.001). (**B**) In vitro cytotoxicity of bulk composite and different composite nanocrystals after 24 h co-incubation (n = 6, mean ± SD). Tested cell line: drug-resistant human breast cancer cells MCF-7/ADR.

Intracellular PTX and LAPA concentrations of the bulk composite, cNC, cNC@PDA and cNC@PDA-PEG were 409.49 ± 6.98 ng, 754.63 ± 3.29 ng, 1246.16 ± 3.18 ng and 3165.85 ± 7.62 ng per 10^6^ cells after incubation, respectively (Figure 8A). Higher intracellular uptake of PTX and LAPA leads to higher cytoxicity. As was shown in Figure 8B, the cytotoxicity was assessed by introducing various concentrations of the binary mixture of bulk PTX and LAPA and the nanocrystals. After incubation with the bulk composite, cNC, cNC@PDA and cNC@PDA-PEG, IC_50_ was 456.40, 293.70, 134.80 and 58.54 nM, respectively. Obviously, cNC@PDA-PEG exhibited the strongest antiproliferative effect of tumor cells. By contrast, cNC did not show any obvious inhibition on the growth of tumor cells, which was consistent with its low cellular uptake.

### 2.5. cNCs Entered Cells in an Intact Form

**Figure 9:**
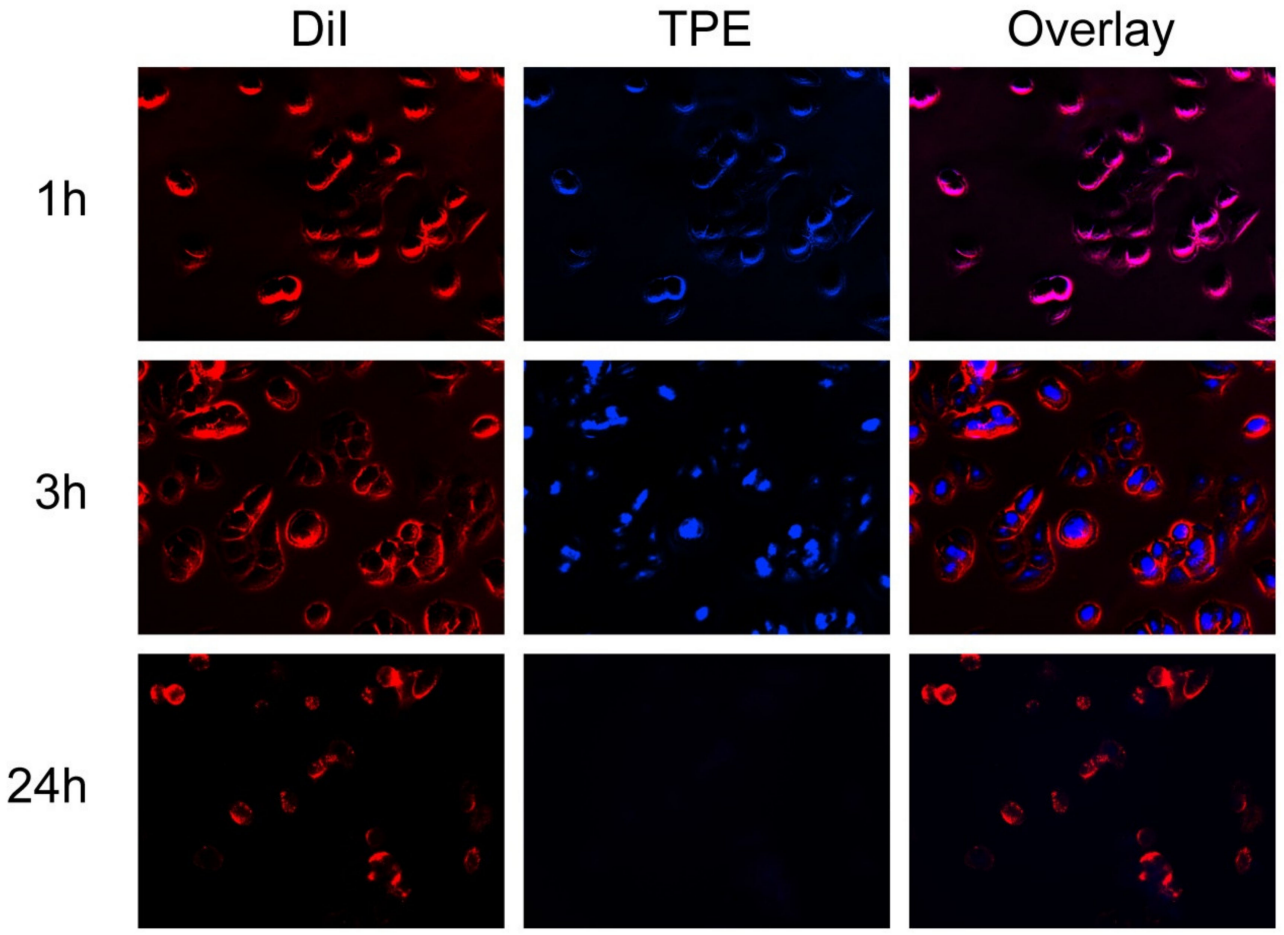
Fluorescent microscopic images of MCF-7/ADR cells cultured with tetraphenylethylene (TPE)-labeled cNC@PDA-PEG for 1, 3 and 24 h. TPE is shown in blue and cell membranes in red. TPE emitted fluorescence when nanocrystals were intact, and would not emit when nanocrystals dissolved. Excitation: 350 nm, emission: 450 nm.

Although the carrier-free nanocrystals have a good anti-tumor effect, it was not clear how drug nanocrystals exert their anticancer effect on cancer cells. Did cNC@PDA-PEG enter cells in an intact form? In 2017, Wei G et al. resorted to aggregation-induced emission (AIE) and developed composite nanocrystals that integrated with AIE fluorophores in order to characterize the dissolution kinetics of the nanocrystals inside cells and eventually in animal models [39]. In the literature, tetraphenylethylene (TPE) was used as a probe to mix with the raw materials for the preparation of nanocrystals to form composite nanocrystals. TPE would emit fluorescence when nanocrystals were excited, and when nanocrystals dissolved, drugs and TPE would release, and the fluorescence intensity would decrease. Before cell experiments, the AIE feature of TPE was verified in ethanol and water mixtures of various mixing ratios. Once completely dissolved in ethanol, TPE started to precipitate when water was introduced to the solution due to the extremely low solubility of TPE in water. As shown in Figure S4, the addition of ethanol caused the TPE-labeled cNC@PDA-PEG to dissolve and released TPE, which in dissolved form lost its fluorescence-emitting feature. The uptake of TPE-labeled cNC@PDA-PEG in MCF-7/ADR cells was observed by inverted fluorescence (Figure 9). The image showed that most of the TPE-labeled cNC@PDA-PEG was co-located with the cell membrane after 1 h, indicating that the cells had just begun to absorb TPE-labeled cNC@PDA-PEG. By 3 h, blue fluorescence appeared in the cells, indicating that the TPE-labeled cNC@PDA-PEG had been internalized by the cells. At 24 h, the fluorescence intensity almost completely disappeared, indicating the dissolution (and possible exocytosis) of the nanocrystals. It was very likely that at least a proportion of TPE-labeled cNC@PDA-PEG entered the cells in the intact form of nanocrystals and then disintegrated in the cells with elapsed time.

## 3. Materials and Methods

### 3.1. Materials

PTX, LAPA, D-alpha-Tocopheryl polyethylene glycol 1000 succinate (TPGS), citric acid, dopamine hydrochloride, 1,1-dioctadecyl-3,3,3,3-tetramethy-lindodicarbocyanine perchlorate (DiD), 1,1′-dioctadecyl-3,3,3′,3′-tetramethy-lindocarbocyanine perchlorate (DiI), 2-(4-amidinophenyl)-6-indolecarbamindine dihydrochloride (DAPI) and 6-coumarin (C6) were purchased from Meilun Biotechnology Ltd. Co. (Dalian, China). 1,1,2,2-tetraphenylethylene (TPE) (>98% purity) was bought from Tixiai Huacheng Industrial Development Ltd. Co. (Shanghai, China). Mal-PEG3000-NH_2_ was purchased from JenKem Technology Ltd. Co. (Beijing, China). All other chemicals were of analytical grade, purchased from Sinopharm Reagent Ltd. Co. (Shanghai, China) and used as received. 

The drug-resistant human breast cancer cell line MCF-7/ADR was purchased from KeyGen BioTECH (Shanghai, China) and cultured in a Roswell Park Memorial Institute 1640 Medium (Gibco, Thermo Fisher Scientific, Waltham, MA, USA) supplemented with 10% fetal bovine serum (FBS) (Gibco, Thermo Fisher Scientific, Waltham, MA, USA), 100 U/mL penicillin and 100μg/mL streptomycin at 37 °C in a 5% CO_2_/95% air humidified atmosphere. Digestive cells were digested with EDTA (Ethylenediaminetetraacetic acid) trypsin digestive juice without phenol red. All centrifugation in this research was performed by a centrifugal machine H1650-W (XiangYi Instruments Ltd. Co., Changsha, China).

### 3.2. Preparation of cNC, cNC@PDA and cNC@PDA-PEG

First of all, the best intracellular drug ratio was determined, based on MTT in MCF-7/ADR cell lines. MCF-7/ADR cells were incubated with PTX (0.075 mg/mL) and LAPA at different concentrations (i.e., the mass ratios of paclitaxel to lapatinib was 10:1, 2:1, 1:1, 1:2.5, 1:5 and 1:10). The intracellular paclitaxel content was detected after incubation for 4 h. The ratio of PTX to LAPA was further optimized using a cell proliferation experiment. Keeping the total mass of the two drugs unchanged (3.0 mg), the six mass ratios of PTX:LAPA (10:1, 2:1, 1:1, 1:2.5, 1:5, 1:10) and the single PTX and single LAPA group were set. The above eight groups were weighed and dissolved in DMSO, diluted to an appropriate concentration and incubated with MCF-7/ADR cells; the MTT method and intracellular concentration quantitative method were used to select the best proportion. The results of the MTT experiment were calculated by Graph Pad software, and the synergy of the two drugs was judged by a combined action index (combined index, CI). CI_50_ >1, =1, and <1 indicated synergistic, additive and antagonistic effects, separately. CI_50_ = DA/IC_50,A_+DB/IC_50,B_ (A, B stands for two different drugs). IC_50,A_ and IC_50,B_ are the IC_50_ when the two drugs are used alone. DA and DB are the IC_50_ when two drugs are used in combination [40].

Formulation optimization should consider not only the efficacy at the cell level, but also the actual preparation. We chose the bottom-up method, which has the advantages of simple preparation and instrument requirements. A mixture of different ratios of PTX and LAPA (a total of 3.0 mg) and stabilizer (4.0 mg D-alpha-Tocopheryl polyethylene glycol 1000 succinate (TPGS)) were dissolved in ethanol. A thin film was formed with a rotary evaporator and then hydrated by deionized water. Citric acid (7.5 mg) was dissolved in ethanol to form a film. In the process of hydration, 5 mL 35 mM NaHCO_3_ aqueous solution was used instead of deionized water. The addition of this pair of acids and bases was in order to reduce the size of nanocrystals and provide the weak alkalinity that was beneficial to the self-polymerization of dopamine.

A mixture (3.0 mg) of PTX, LAPA and stabilizers (4.0 mg TPGS and 7.5 mg citric acid) were fully dissolved in 3 mL of ethanol in an eggplant flask. A thin film was formed with a rotary evaporator. 5 mL 35 mM NaHCO_3_ aqueous solution was added for hydration at room temperature to form a composite nanocrystal (cNC). Dopamine (3.7 mg) was added to 5 mL of the above-mentioned cNC suspension and magnetically stirred at room temperature for 12 h. The unreacted dopamine and self-polymerized PDA were removed after centrifugation at 8000 rpm for 10 min at room temperature to form cNC@PDA. Then cNC@PDA were resuspended in 5 mL distilled water containing 3.0 mg MeO-PEG3000-NH_2_ and reacted for 6 h to obtain cNC@PDA-PEG. The suspension was centrifuged at 8000 rpm for 10 min at room temperature to remove unreacted functional materials and obtain cNC@PDA-PEG. C6-labeled cNC, cNC@PDA or cNC@PDA-PEG were similarly prepared, except that in the first step 25 μg C6, PTX and LAPA (total 3.0 mg), and stabilizers (4.0 mg TPGS and 7.5 mg citric acid) were added to 3 mL of ethanol in an eggplant flask. TPE-labeled cNC@PDA-PEG was also similarly prepared, except that in the first step extra TPE (0.06 mg) was added to 3 mL of ethanol in an eggplant flask.

### 3.3. Characterization

#### 3.3.1. Particle Size and Distribution

Different nanocrystals were put into sample cells (a sample cell specially used for particle size or zeta potential measurement), and then the cell was inserted into a Malvern instrument. After temperature equilibrium, it was measured at 37 °C by dynamic light scattering (DLS) using a Zetasizer (ZS-10-82, Malvern Instruments Ltd. Co., Malvern, UK).

#### 3.3.2. Morphology

The morphology of different nanocrystals was observed by transmission electron microscopy (TEM) on a Tecnai G2 F20 S-TWIN (FEI Co., Hillsboro, Oregon State, USA). First, 20 μL of particle solution at a total drug concentration of 60 μg/mL was dropped onto a glow-discharged carbon-coated grid. After 10 min, the sample was blotted, and then the grid was subsequently dried.

#### 3.3.3. Drug Loading (DL) and Encapsulation Efficiency (EE)

Freeze-dried nanocrystals with a premeasured mass were dissolved in an appropriate volume of acetonitrile (ACN) and filtered with a 0.45 μm syringe filter prior to analysis. The solution was diluted if necessary before it was submitted to HPLC analysis on an Agilent 1100 HPLC system (Palo Alto, CA, USA). The PTX was quantified on a Diamonsil^®^C18 column (250 mm × 4.6 mm:5 μm, Beijing, China) with a mobile phase of 70:30 ACN:H_2_O (*v*/*v*) at a flow rate of 0.7 mL/min and a detection wavelength of 227 nm. The LAPA was quantified on a Xtimate^®^C18 column (250 mm × 4.6 mm:5 μm, Shanghai, China) with a mobile phase of 65:35 ammonium acetate:water (*v*/*v*) at a flow rate of 0.7 mL/min and a detection wavelength of 272 nm. Drug loading (DL) and encapsulation efficiency (EE) are calculated according to the following equations, respectively.

(1) $$\mathrm{EE}\;(\%)\;=\;\frac{\mathrm{Amount}\;\mathrm{of}\;\mathrm{drug}\;\mathrm{in}\;\mathrm{nanocrystals}}{\mathrm{Total}\;\mathrm{amount}\;\mathrm{of}\;\mathrm{feeding}\;\mathrm{drug}}\;\times \;100\%$$

(2) $$\mathrm{DL}\;(\%)\;=\;\frac{\mathrm{Amount}\;\mathrm{of}\;\mathrm{drug}\;\mathrm{in}\;\mathrm{nanocrystals}}{\mathrm{Total}\;\mathrm{amount}\;\mathrm{of}\;\mathrm{nanocrystals}}\;\times \;100\%$$

#### 3.3.4. X-ray Powder Diffraction (XRPD) and Differential Scanning Calorimeter (DSC)

X-ray powders diffraction (XRPD) of different nanocrystals was analyzed with a D2 Phaser diffractometer (BrukerCorp, Billerica, Massachusetts) with a Cu-Kα radiation source and a LYNXEYETM-compound silicon strip detector. The powder patterns were obtained from 0 to 50° 2θ at a scan speed of 5°/min and a step size of 0.02°. The voltage and current used were 40 kV and 100 mA, respectively. Differential scanning calorimeter (DSC) analysis of different nanocrystals was measured by a Perkin–Elmer Pyris 1 DSC instrument (Waltham, MA, USA) with an Intra-cooler 2P cooling accessory. The pre-weighed samples were sealed into standard aluminum pans and scanned from 0 to 500 ℃ at a heating rate of 10 ℃/min with a nitrogen purge of 10 mL/min, respectively.

#### 3.3.5. Stability

The stability of different composite nanocrystals was evaluated in phosphate buffer saline (PBS) and fetal bovine serum (FBS). Because FBS may affect the measurement of size, a previously reported method was used to monitor the agglomeration of composite nanocrystals in its presence. Particle size was measured by dynamic light scattering (DLS) in PBS and the absorbance in both PBS and 10% FBS was measured by a Synergy 2 enzyme labeling instrument (Biotek, Green Mountains, Vermont, USA) at 560 nm [41].

#### 3.3.6. In Vitro Release

The drug release curves of different nanocrystals in 0.1 mM PBS (pH 7.4) were measured using the dialysis bag method [42]. In short, in the dialysis bag (MWCO = 35,000 Da; Thermo Fisher Scientific, Waltham, MA, USA) different nanocrystals of 1 mL (concentration of PTX equivalent to 400 μg/mL, concentration of LAPA equivalent to 200 μg/mL) were added, and then the dialysis bag was completely immersed in the release medium of 10 mL containing 0.1% Tween-80 (*w*/*w*). The release experiment was carried out at 37 °C in a reciprocating vibrating shaking machine at a speed of 100 rpm (THZ-103B, Shanghai Hengyi Scientific Instruments Co. Ltd. Shanghai, China). The concentrations of PTX and LAPA in the release medium were determined by HPLC under similar conditions to Section 3.3.3.

#### 3.3.7. Hemolysis Assay

The hemolysis of red blood cells was measured to evaluate the blood compatibility of different nanocrystals. Fresh blood samples from male Sprague–Dawley rats were taken through the retroorbital venous plexus. The blood was collected in an anticoagulant tube containing heparin sodium, and then the sample was centrifuged with 1500 rpm for 10 min and washed with PBS three times. The supernatant was discarded and the erythrocyte suspension was prepared with PBS. Different composite nanocrystals with final concentrations of 0.06, 0.12, 0.18, 0.24 and 0.3 mg/mL were incubated with a red blood cell suspension at 37 °C for 2 h and 6 h. Saline and water were set as negative and positive controls, respectively. Finally, the supernatant was obtained by centrifugation under 1500 rpm for 10 min, and its absorbance at 541 nm was measured by an enzyme labeling instrument to check the blood compatibility of the sample.

### 3.4. Cellular Experiments

#### 3.4.1. Cellular Uptake

For fluorescent images, MCF-7/ADR cells were seeded in a 6-well plate at the density of 2 × 10^5^ cells per well. After overnight incubation, the medium was replaced by fresh medium, to which the C6-labeled cNC, cNC@PDA and cNC@PDA-PEG were added to obtain the final concentration of C6 equivalent to 1 nM. After incubation for 4 h, the medium was removed and the cells were washed with PBS twice in order to wash away the unbound composite nanocrystals. The cells were fixed with 4% paraformaldehyde in PBS for 15 min, and the nuclei were stained with DAPI. Fluorescent images were taken with a fluorescence microscope (Leica, DMI4000 B, Frankfurt, Germany).

For co-localization with lysosomes, the cells which had been incubated with various composite nanocrystals for 4 h were treated with a LysoTracker Red DND-99 (25 nM) for 30 min and fixed with 4% paraformaldehyde in PBS for 15 min, followed by a 5 min treatment of DAPI to stain the nucleus of the cells. Fluorescent images were taken with a Leica confocal microscope (Leica, TCS SP8, Frankfurt, Germany).

For flow cytometry (FCM), MCF-7/ADR cells were similarly seeded in 6-well plates and co-incubated with C6-labeled cNC, cNC@PDA and cNC@PDA-PEG, except that the final concentration of C6 equivalent was 10 nM. After incubation for 4 h, the medium was removed and the cells were washed with PBS twice to wash away unbound composite nanocrystals. The cells were then collected by centrifugation at 1000 rpm and resuspended in PBS. The fluorescence labeled cells were counted by a flow cytometer BD FACSCalibur Flow Cytometry System (Applied Cytometry Systems, NJ, USA) at Ex 466 nm and Em 504 nm.

For quantitative comparison of intracellular drug content, MCF-7/ADR cells were seeded in 6-well plates at a density of 2 × 10^5^ cells per well. After overnight incubation, the medium was replaced by fresh medium, to which cNC, cNC@PDA and cNC@PDA-PEG were added to obtain the final concentration of PTX equivalent to 20 μg/mL. After incubation of the treatment for 4 h, the medium was removed and the cells were washed with PBS two times to wash away unbound composite nanocrystals. The cells were then collected by centrifugation at 1000 rpm and resuspended in PBS:CAN = 1:1 (*v*/*v*). The cells were lysated by ultrasonic for 10 min in a water bath. The content of PTX was measured by HPLC under similar conditions as in Section 3.3.3.

#### 3.4.2. In Vitro Cytotoxicity

MCF-7/ADR cells were seeded in 96-well plates at a density of 3000 cells per well. After overnight incubation, the medium was replaced by fresh medium, to which free PTX and LAPA (bulk composite), cNC, cNC@PDA and cNC@PDA-PEG were added to provide the final concentration of PTX equivalent to 7.94 nM, 6.31 nM, 5.25 nM, 3.99 nM, 2.64 nM, and 2.00 nM, respectively. After a 24 h incubation, the 3-(4,5-dimethylthiazol-2-yl)-2,5-diphenyltetrazolium bromide) (MTT) assay was adopted to measure the cell viability. Briefly, MCF-7/ADR cells were treated with MTT (5 mg/mL, 20 μg per well) and incubated for 4 h. The medium was removed and formazan crystals were dissolved in dimethyl sulfoxide (DMSO). Quantification analysis was performed by a Power Wave XS Microplate Spectrophotometer (BioTek Instruments, Inc. Green Mountains, Vermont, USA). PTX and LAPA were dissolved in DMSO to prepare the bulk composite.

#### 3.4.3. AIE Experiment

In order to answer the question “was the nanocrystal taken up by the cells intactly or not”, tetraphenylethylene (TPE), an aggregation-induced emission (AIE) fluorescent probe, was used to label composite nanocrystals. Such probes emit fluorescence only when the probe molecules are close enough to each other. Hence it could be expected that TPE-labeled composite nanocrystals could emit fluorescence only in the intact form [39]. MCF-7/ADR cells were inoculated in 6-well plates at a density of 1 × 10^5^ cells/well, and then incubated with TPE-labeled cNC@PDA-PEG for 1, 3 and 24 h. The final concentration of TPE was 50 μM. At the end of incubation, the cells were gently washed with cold PBS three times and DiI (25 nM) was added. Cells were incubated at 37 °C for another 20 min. Then cells were gently washed with cold PBS three times, fixed with 4% paraformaldehyde in PBS at room temperature for 10 min, and washed with cold PBS three times. Fluorescence images of the cells were taken by a Leica fluorescence microscope (Leica, DMI4000 B, Frankfurt, Germany).

## 4. Conclusions

In conclusion, PEGylated PTX and LAPA composite nanocrystals (cNC, 2:1 (*w*/*w*)) were successfully prepared. cNC@PDA-PEG had a lower hemocytolysis effect in vitro and enhanced therapeutic effects on MCF-7/ADR cells compared with the unmodified cNC. Such composite nanocrystals of PTX and LAPA may provide a potential formulation for treatment of chemo-resistant cancer in future.

## Figures and Tables

**Table 1:** IC_50_ and CI_50_ values of the PTX/LAPA combination against MCF-7/ADR cells (24 h) (n = 6, mean ± SD). CI: combined index.

Treatment (P:L)	IC_50_ of PTX (nM)	IC_50_ of LAPA (nM)	CI_50_
PTX	6965.00		
10:1	1427.00	314.5	0.23
2:1	733.80	399.41	0.14
1:1	860.40	1264.00	0.22
1:2.5	807.8	2954.00	0.35
1:5	1151.00	8455.00	0.84
1:10	2141.00	22090.00	2.07
LAPA		12510.00	

**Table 2:** Drug loading (DL) and encapsulation efficiency (EE) of different composite nanocrystals (n = 3, mean ± SD). PDA: polydopamine.

Formulation	DL (PTX)	EE (PTX)	DL (LAPA)	EE (LAPA)
cNC	32.35 ± 1.48	45.29 ± 2.42	15.31 ± 1.57	22.65 ± 3.14
cNC@PDA	25.38 ± 1.56	48.22 ± 1.96	13.33 ± 1.43	24.11 ± 1.86
cNC@PDA-PEG	21.33 ± 1.43	54.60 ± 1.39	10.95 ± 1.24	27.30 ± 2.33

## Supplementary Materials

The following are available online at https://www.mdpi.com/1420-3049/25/3/604/s1. Figure S1: Cytotoxicity of free PTX incubated with MCF-7 or MCF-7/ADR for 24 h. RI = IC_50_ drug resistant cell/IC_50_ pre-induction cell. (n = 3, mean ± SD); Table S1: Optimization of paclitaxel and lapatinib with different ratios (n = 3, mean ± SD); Figure S2: (A) Optimizing the size and PDI of the formulation by changing the ratio of paclitaxel to lapatinib (n = 3, mean ± SD). Tyndall effect of freshly prepared nanocrystals (B) and placed for 5 min (C); Figure S3: Size aggregation in PBS was measured by absorbance at 560 nm; Figure S4: Glass vials of TPE NC and TPE-labeled cNC@PDA-PEG in water/ethanol mixtures of various *v/v* ratios under UV illumination.
