# Supplementary material for: Synergistic Antitumor Effects on Drug-Resistant Breast Cancer of Paclitaxel/Lapatinib Composite Nanocrystals
Source: Molecules. 2020 Jan 30;25(3):604. doi: 10.3390/molecules25030604 (PMC7036807; doi:10.3390/molecules25030604)
Supplement: Supplementary file 1 [file molecules-25-00604-s001.pdf]

molecules MDPI

Supplementary Information: Considering the results of preparation, when PTX was used alone to form PTX NC, the particle size was 79.6±1.6nm, PDI was 0.14±0.01. When LAPA were added to form P:L=10:1, the particle size was 87.7 ±1.5nm, PDI was 0.12±0.01. With the increase of the concentration of LAPA, the particle size and PDI increased. When LAPA increased to form P:L=1:1, the particle size was 175.3±2.5, PDI was larger than 0.2. When there was LAPA only, the particle size was 6610.7±332.9 nm, and the PDI was 0.89±0.16 (Table S1). The results showed that the particle size increased with the increase of LAPA. The results of the Tyndall effect showed that the beam passed through PTX NC, P:L=10:1 and 2:1, and the scattering occurred in P:L=1:1, which might be precipitation (Figure S2B). After 5min placement, the beam still passed through PTX NC, P:L=10:1 and 2:1, while more obvious than the fresh one in P:L=1:1, which might be the precipitation fell to the bottom of the bottle. P:L=1:5, 1:10 and LAPA NC produced more precipitation (Figure S2C). Combined with the above results, we chose P:L=2:1.

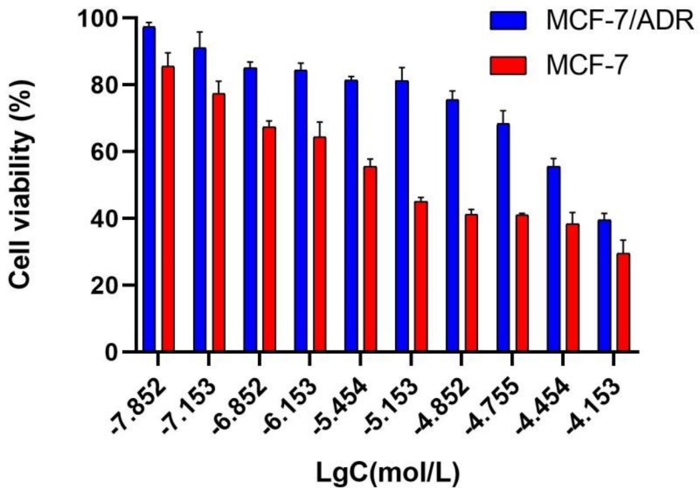

Figure S1. Cytotoxicity of free PTX incubated with MCF-7 or MCF-7/ADR for 24 h. RI= IC_{50} drug resistant cell/IC_{50} pre-induction cell. (n = 3, mean ±SD).

Table S1. Optimization of paclitaxel and lapatinib with different ratios (n=3, mean ± SD).

Formulation (P:L)	Size (nm) ± SD	PDI ±SD
PTX NC	79.6±1.6	0.14±0.01
10:1	87.7±1.5	0.12±0.01
2:1	95.1±1.1	0.14±0.02
1:1	175.3±2.5	0.24±0.03
1:2.5	220.4±3.1	0.30±0.02
1:5	327.6±4.4	0.65±0.14
1:10	645.3±8.9	0.66±0.03
LAPA NC	6610.7±332.9	0.89±0.16

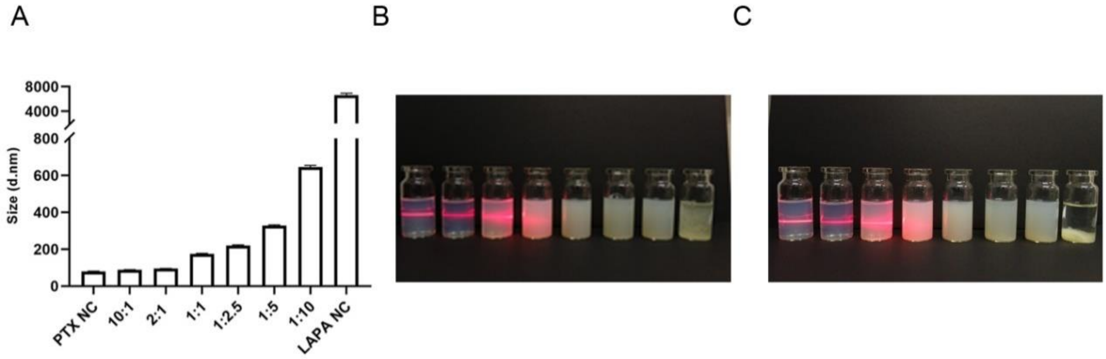

Figure S2. (A)Optimize the size and PDI of the formulation by changing the ratio of paclitaxel to lapatinib (n = 3, mean ± SD). Tyndall effect of freshly prepared nanocrystals (B) and placed for 5 min (C).

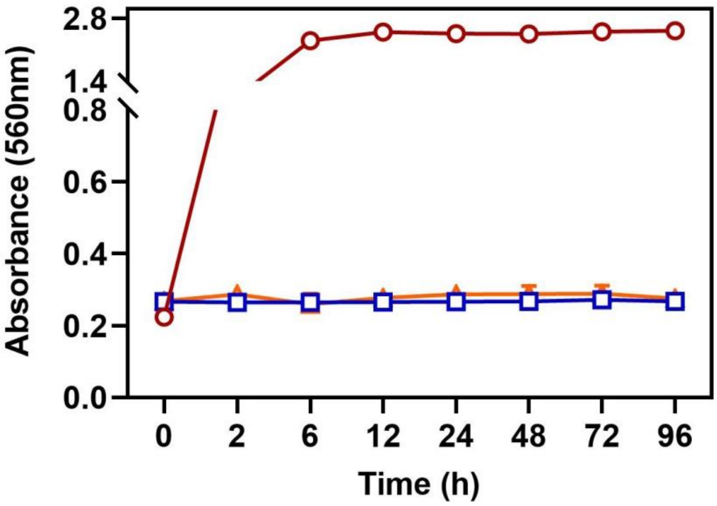

Figure S3. Size aggregation in PBS measured by absorbance at 560 nm.

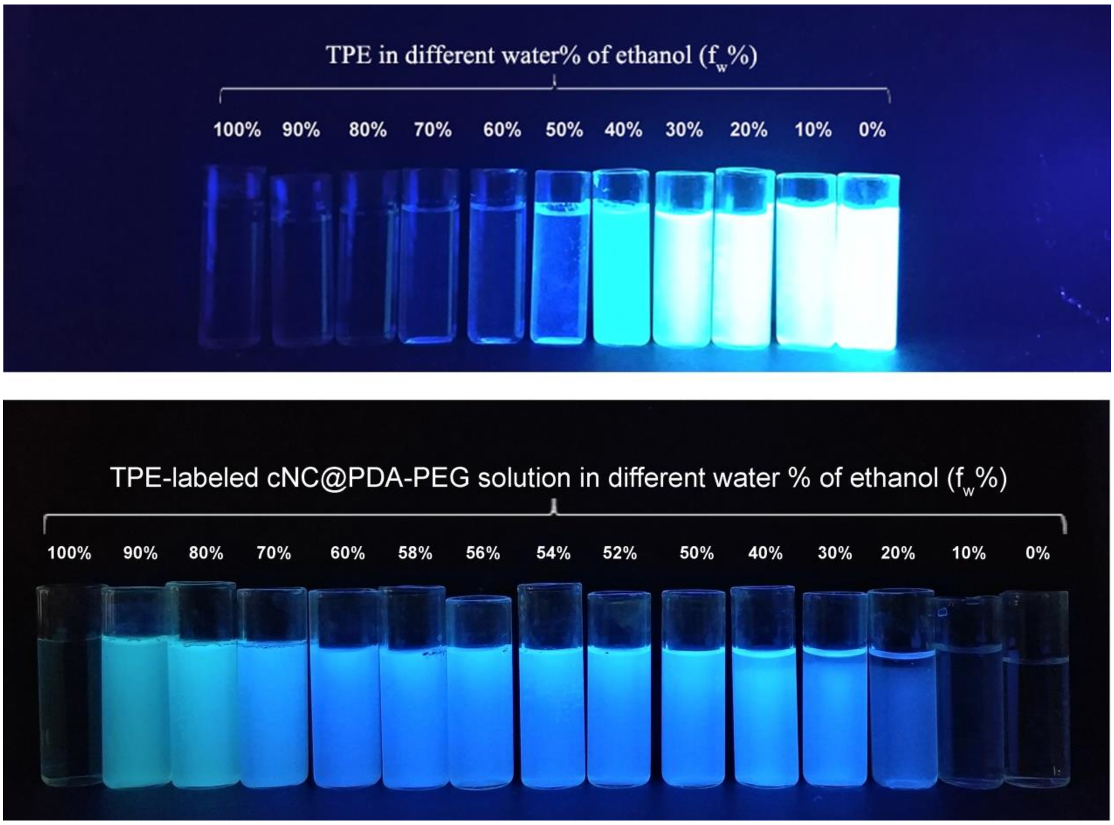

Figure S4. Glass vials of TPE NC and TPE-labeled cNC@PDA-PEG in water/ethanol mixtures of various v/v ratios under UV illumination.
